# Detection of multidrug-resistant *Acinetobacter baumannii* by metagenomic next-generation sequencing in central nervous system infection after neurosurgery: A case report

**DOI:** 10.3389/fpubh.2022.1028920

**Published:** 2022-10-21

**Authors:** Ying Tian, Han Xia, Linlin Zhang, Jian-Xin Zhou

**Affiliations:** ^1^Department of Critical Care Medicine, Beijing Tiantan Hospital, Capital Medical University, Beijing, China; ^2^Department of Scientific Affairs, Hugo Biotechnologies, Co., Ltd, Beijing, China; ^3^Beijing Engineering Research Center of Digital Healthcare for Neurological Diseases, Beijing, China; ^4^Beijing Shijitan Hospital, Capital Medical University, Beijing, China

**Keywords:** *Acinetobacter baumannii*, central nervous system infections, metagenomic next-generation sequencing, cerebrospinal fluid, resistance genes

## Abstract

**Background:**

Central nervous system (CNS) infection is one of the most serious complications after neurosurgery. Traditional clinical methods are difficult to diagnose the pathogen of intracranial infection. Due to recent advances in genomic approaches, especially sequencing technologies, metagenomic next-generation sequencing (mNGS) has been applied in many research and clinical settings.

**Case presentation:**

Here, we report a case of CNS infection with *Acinetobacter baumannii* in a 15-year-old woman, who previously underwent surgery for recurrence of ependymoma in the fourth ventricle. On the eleventh postoperative day, the patient had a high fever and leukocytosis in the cerebrospinal fluid (CSF). mNGS using CSF rapidly and accurately identified the causative pathogen as *A. baumannii* with carbapenem resistance genes *blaOXA-23* and *blaOXA-51*, which were confirmed by subsequent culture and susceptibility tests within 5 days. During the disease, mNGS, culture, and drug susceptibility testing were continued to monitor changes in pathogenic bacteria and adjust medication. At present, there are no case reports on to the use of mNGS for detecting pathogens in postoperative infection with ependymoma and guide medication.

**Conclusion:**

mNGS has great advantages in pathogen identification and even pathogen resistance prediction. Multiple mNGS examinations during the course of the disease play an important role in the dynamic monitoring of pathogens.

## Background

Central nervous system (CNS) infection is caused by pathogenic microorganisms invading the CNS, manifesting as a class of inflammatory or non-inflammatory diseases with acute or chronic symptoms. The pathogenic microorganisms of CNS infection mainly include viruses, bacteria, fungi, and parasites. CNS infections can be community-acquired or hospital-acquired. Patients with a history of neurosurgery, severe neurotrauma, or indwelling cerebrospinal fluid (CSF) drainage experience a high risk for hospital-acquired CNS infections (1). Despite current advances in clinical diagnosis and treatment, CNS infections still have a high morbidity and mortality, with an estimated 320,000 deaths due to meningitis worldwide in 2016 (2).

Metagenomic next-generation sequencing (mNGS) technology, capable of deciphering millions of DNA and RNA sequences in parallel, has shown promise for detecting pathogens in clinical samples (3). A major advantage of mNGS is the unbiased simultaneous detection of multiple pathogens, its broad identification of known and unexpected pathogens, and the discovery of new organisms (4). The unbiased nature of mNGS aids neurologists in evaluating complex clinical cases with incomplete information. Another advantage of mNGS is that it can provide auxiliary genomic information needed for evolutionary tracking, strain identification, and resistance prediction (5–8). mNGS can quantify or semi-quantify the concentration of organisms in a sample, which is useful for multiple microbial samples or when more than one pathogen is involved in the disease process (9).

*Acinetobacter baumannii* (*A. baumannii*) is a non-enteric gram-negative bacillus characterized by low virulence. As an opportunistic nosocomial pathogen, *A. baumannii* has been one of the most important multidrug-resistant (MDR) microorganisms in hospitals worldwide. This human pathogen causes a variety of infections, of which ventilator-associated pneumonia and bloodstream infections are the most common, with a mortality rate of 35% (10). *A. baumannii* associated CNS infection is common in patients with brain surgery and extra ventricular drainage through a catheter (11). The ability of *A. baumannii* to develop resistance to currently used antibiotics is quite high compared to other bacteria (12). Intrathecal or intracerebroventricular administration of colistin has become an increasingly common approach for the treatment of MDR or extensive drug-resistant (XDR) *A. baumannii* associated CNS infections (13–15).

## Case presentation

Here, we report a 15-year-old female patient who was presented to a local hospital in 2018 due to headache and vomiting (Supplementary Figure 1). Magnetic resonance imaging (MRI) showed an intracranial occupying lesion. Subsequently, she underwent a quadruple ventricular tumor resection in a Beijing hospital. The pathology report of the tumor was WHO grade II ventricular meningioma. The patient recovered well after the operation.

**Figure 1:**
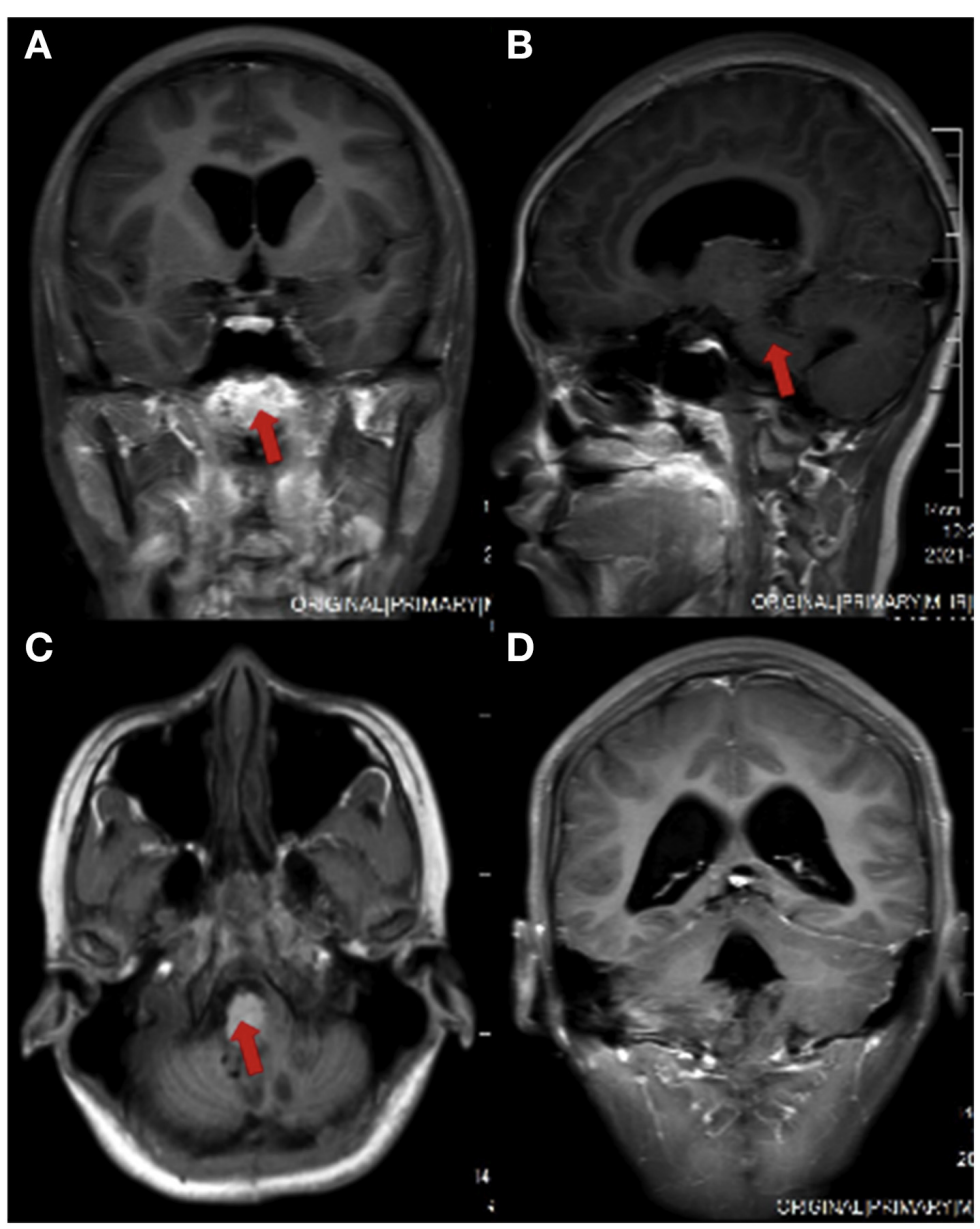
Head MRI of the patient before surgery. **(A)** coronal view; **(B)** sagittal section view; **(C)** axial view; **(D)** ventricular enlargement in coronal view

However, in June 2021, the patient developed pulsating headaches with dizziness and vomiting again, and the symptoms gradually worsened. Therefore, she came to Beijing Tiantan Hospital for further treatment. MRI (Figure 1) of the head at our hospital showed bilateral cerebellopontine angle (CPA) and fourth ventricle occupying lesions with a high likelihood of ventricular meningioma recurrence and hydrocephalus. After a series of preoperative examinations, the patient underwent a posterior median craniotomy and artificial dural repair under general anesthesia. Postoperatively, she was kept in the intensive care unit with tracheal intubation and mechanical ventilation to assist breathing, followed by airway care, hormones, dehydration, rehydration, and symptomatic treatment.

**Figure 2:**
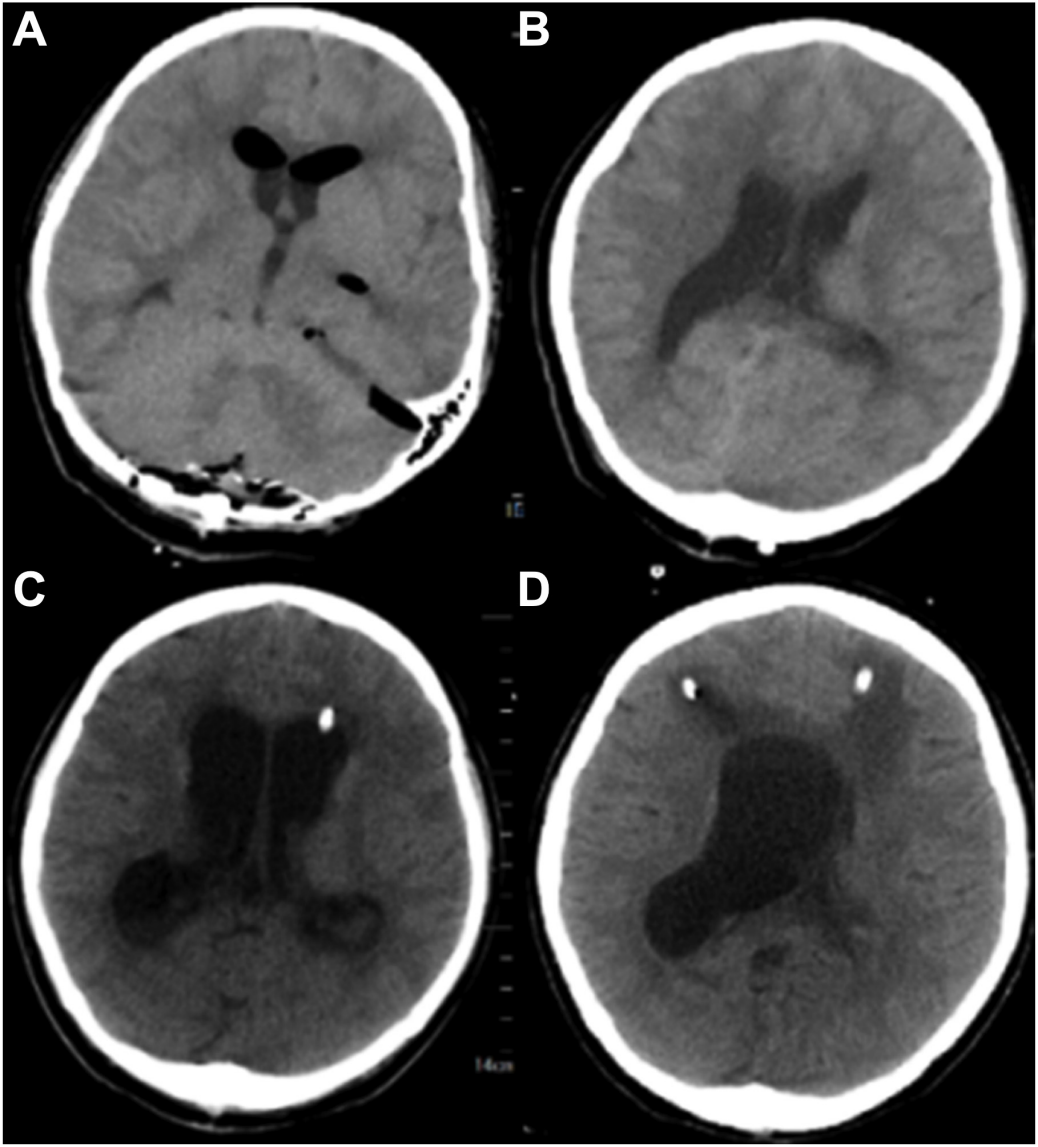
Brain CT of the patient in different stages of the disease. **(A, B)**: Postoperative CT of the patient. **(C)** On the 17th postoperative day, the patient's head CT after left ventriculocentesis. **(D)** On the 27th postoperative day, the patient's head CT after left ventriculocentesis.

Postoperative cranial Computed Tomography (CT) showed a “post-occipital craniotomy” status, enlargement of the ventricular system, and a slight intracerebroventricular hyperintensity (Figures 2A,B). The patient was conscious on the first postoperative day, with a temperature of 39°C, absolute white blood cell (WBC) value of 20.99 × 10^9^/L, and granulocyte (GR) value of 96.9%. Considering that the elevated body temperature and absolute WBC values might be related to postoperative stress, the patient was given physical cooling and anti-infective treatment with cefuroxime (1.5 g, q12h). After symptomatic treatment, the patient's vital signs were stable, the temperature dropped to 36.5°C, and the inflammatory indexes gradually decreased to normal levels on the sixth postoperative day. Therefore, ventilator withdrawal was attempted on the sixth postoperative day, and transoral tracheal intubation was removed, but the patient's blood oxygen level decreased, and her respiratory rhythm was irregular. So, transnasal tracheal intubation with continuous T-tube oxygenation was performed again.

**Figure 3:**
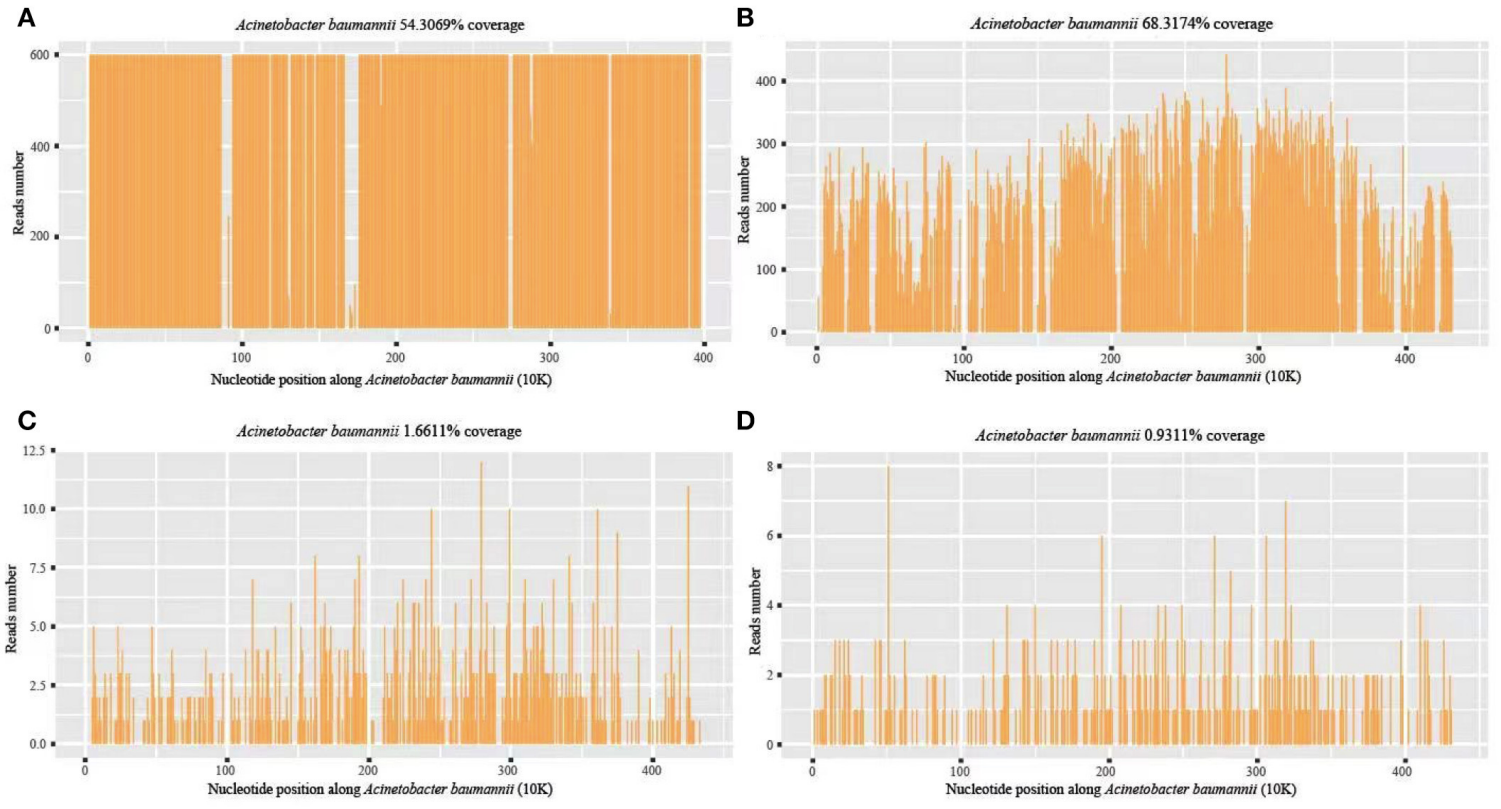
*A. baumannii* coverage map of mNGS detection in different periods. **(A)** The first mNGS of CSF: 99,343 specific sequences of *A. baumannii*, with a coverage of 54.3%; **(B)** The second mNGS of CSF: 88,618 specific sequences and 68.32% coverage; **(C, D)** The third mNGS of CSF and blood: 857 (coverage 1.66%) and 446 (coverage 0.93%) specific reads, respectively.

On the eleventh postoperative day, the patient's consciousness was clear, but the body temperature rose to 39.5°C, with the pulse rate of 130 beats/min and WBC of 33.08 × 10^9^/L. Infection of the patient was considered. CSF and blood were drawn from the patient for routine testing and cultures. CSF routine results revealed orange-red, cloudy CSF in appearance with total cells of 3,585/μl, pericytes of 1,085/μl, multinucleated cells ratio of 97.9%, mononuclear cells ratio of 2.1%, glucose of 0.68 mmol/L, protein of 413.6 mg/dL, chloride of 106 mmol/L, and Lactate of 18.6 mmol/L. The presence of intracranial infection in the patient could not be excluded. The patient was given an antibiotic regimen of vancomycin hydrochloride (1000 mg, q12h) and meropenem (2 g IV q8h). On the twelfth day after surgery, the patient's temperature and pulse decreased (37.5°C and 110 beats/min), but the WBC was still high (64.58 × 10^9^/L). The patient's vital signs were assessed by a neurosurgeon, and then a lumbar pool tube was placed to drain CSF on the same day, which was immediately sent for mNGS by illumina's NextSeq550DX (Hugobiotech, Beijing, China). On the fourteenth postoperative day, the CSF mNGS results revealed 99,343 specific sequences of *A. baumannii*, with a coverage of 54.3% (Figure 3A). Carbapenem resistance genes *blaOXA-23* (36 reads) and *blaOXA-51* (15 reads) were also detected (Table 1). We discontinued vancomycin and added polymyxin (500,000 units, q12h) following the guidelines for the treatment of *A. baumannii* in The Sanford Guide to antimicrobial therapy. The CSF culture and drug susceptibility test on the sixteenth postoperative day confirmed the mNGS results, indicating intracranial reinfection caused by MDR *A. baumannii* (Table 1). Her blood culture was negative. Therefore, we stopped using meropenem and added tigecycline injection (50 mg, q12h), along with intrathecal polymyxin (50,000 units, q12h). The dynamic monitoring of CSF-related indicators continued in this patient.

**Table 1:** Drug resistance results of culture and mNGS.

	**CSF culture (twelfth postoperative day)**		**CSF culture (seventeenth postoperative day)**	
	**Sensitivity**	**MIC**	**Sensitivity**	**MIC**
Piperacillin-tazobactam	R	≥128.0	R	≥128.0
Ceftazidime	R	≥64.0	R	≥64.0
Cefoperazone sulbactam	S	16.0	S	16.0
Cefepime	R	≥32.0	R	≥32.0
Imipenem	R	≥16.0	R	≥16.0
Meropenem	R	≥16.0	R	≥16.0
Tobramycin	R	≥16.0	R	≥16.0
Ciprofloxacin	R	≥4.0	R	≥4.0
Levofloxacin	R	≥8.0	R	≥8.0
Doxycycline	R	≥16.0	R	≥16.0
Minocycline	S	4.0	S	4.0
Tigecycline	S	1.0	S	1.0
Polymyxin	S	≤0.5	S	≤0.5
Compound Sulfonamide	S	≤20.0	S	≤20.0
	CSF mNGS (twelfth postoperative day)	CSF mNGS (seventeenth postoperative day)	CSF mNGS (twenty-seventh postoperative day)	Blood mNGS (twenty-seventh postoperative day)
blaOXA-23	36 reads	56 reads	-	-
blaOXA-51	15 reads	34 reads	-	-
parC	101 reads	98 reads	1 reads	1 reads
gyrA	108 reads	93 reads	-	1 reads
OprD	13 reads	17 reads	-	-
ampC beta-lactamase	23 reads	9 reads	-	-

On the seventeenth postoperative day, the conditions of the patient improved but were still higher than the normal range, with a body temperature of 37.6°C, WBC of 14.19 × 10^9^/L, and GR of 86.4%. CSF routine showed pale yellow and clear in appearance, with total cells of 1137/μl, pericytes of 237/μl, multinucleated cells of 85.6%, mononuclear cells of 14.4%, glucose of 1.19 mmol/L, protein of 100.9 mg/dL, chloride of 119 mmol/L, and lactate of 7.8 mmol/L. To further monitor the progression, mNGS and culture using CSF and blood samples were performed again on the seventeenth postoperative day. The second CSF mNGS result still revealed *A. baumannii* with 88,618 specific sequences and 68.32% coverage (Figure 3B). Besides, drug-resistant genes, including *blaOXA-23* (56 reads), *blaOXA-51* (34 reads), *parC* (98 reads), *gyrA* (93 reads), *oprD* (17 reads) and *ampC* beta-lactamase (9 reads), were detected, suggesting they might resist to both fluoroquinolones and carbapenems, consistent with the results of subsequent CSF culture (Table 1). By contrast, mNGS and culture of blood were negative. Therefore, the current treatment regimen was continued.

A left ventricular puncture and drainage procedure was performed due to the progressive hydrocephalus (Figure 2C) on the seventeenth postoperative day. Postoperative monitoring showed the patient's body temperature, blood routine, CSF routine, and biochemistry were slightly better than before. However, right ventricular puncture and drainage was performed on the twenty-seventh postoperative day due to suspected hydrocephalus according to head CT, which showed an enlarged right ventricle (Figure 2D). The patient's temperature was 37.8°C, with WBC of 12.36 × 10^9^/L and GR of 74%. Right ventricular drainage CSF routine revealed yellowish in appearance, cloudy in nature, total cells of 2231/μl, pericytes of 331/μl, multinucleated cells of 71.3%, mononuclear cells of 28.7%, glucose of 3.07 mmol /L, protein of 363.19 mg/dL, chloride of 115 mmol/L, and lactate of 4.6 mmol/L. The third mNGS using blood and right ventricle CSF was performed on the twenty-seventh postoperative day, revealing 857 (coverage 1.66%) and 446 (coverage 0.93%) specific reads of *A. baumannii*, respectively. Drug-resistant genes (*gyrA* by CSF, and *parC* and *gyrA* by blood, all were 1 sequence) were also detected by both blood and CSF mNGS (Figures 3C,D). The current anti-infective treatment regimen continued.

Two days later, the patient was transferred to a local hospital according to her family's request. She received conservative treatment and was discharged with stable vital signs. The patient was followed up 3 months after discharge with a Glasgow Outcome Scale (GOS) score of 4.

## Discussion and conclusions

Ventricular meningioma is a glioma with ventricular meningeal cell differentiation that can occur in the ventricles and spinal cord. It can be classified into three grades according to 2016 WHO classification criteria, including subventricular and mucinous papillary ventricular meningiomas (grade I), ventricular meningiomas (grade II), and mesenchymal ventricular meningiomas (grade III). Recurrence of primary ventricular meningioma after surgery is common, and younger age, incomplete tumor resection, and high-grade or mucinous papillary ventricular meningioma are the risk factors (16). Surgery for tumors presenting in the fourth ventricle is more challenging, with a probability of postoperative infection of approximately 4.5% (17). The current case is a WHO grade II ventricular meningioma in the fourth ventricle, which recurred 3 years after total tumor resection. There were no metastases elsewhere, but respiratory and circulatory failure and infection after reoperation.

*A. baumannii* is a Gram-negative ESKAPE microorganism with MDR due to widespread antibiotic abuse and mismanagement (18). Immunocompromised and severely ill hosts are susceptible to invasive infections by *A. baumannii*. Carbapenems were once considered the drugs of choice for the empirical treatment of *A. baumannii* infections. However, previous studies have shown that most MDR *A. baumannii* strains (98.1%) were resistant to imipenem, and about 50% were resistant to meropenem, amikacin, and gentamicin. Potent carbapenemase genes were detected in almost all strains, such as *blaOXA-51* (89.3%) and *blaOXA-23* (68.9%) (19). In this case, the patient was in a postoperative state for ventricular meningioma and was vulnerable to XDR *A. baumannii*. The carbapenem resistance gene of *A. baumannii* was detected in the first and second CSF mNGS, suggesting that the strain may be resistant to imipenem and meropenem. And the second and third CSF mNGS also detected the gene encoding DNA gyrase (*gyrA*) and the gene encoding topoisomerase IV (*parC*) at the target site of fluoroquinolone drug action, and mutations at different sites, suggesting that the strain may escape from fluoroquinolones (20). However, these mutations have not yet been reported in the literature on whether they are related to fluoroquinolone resistance. Therefore, we did not adjust the antibiotic treatment plan according to the resistance genes detected by mNGS.

An accurate etiologic diagnosis is important for the treatment of postoperative complications, including serious infections and infections caused by drug-resistant organisms. Raper et al. reported a rare case of herpes simplex virus encephalitis after ventriculotomy, the rapid detection of HSV infection by mNGS played a key role in the timely antiviral treatment of the patient (21). Farrell et al. reported a case of *Enterococcus faecalis* infection detected by polymerase chain reaction (PCR) combined with Electro Spray Ionization-Mass Spectroscopy (ESI-MS) in CSF from a patient with concurrent bacterial meningitis after ventriculoma resection, and emphasized the importance of accurate pathogenic diagnosis and dynamic monitoring of ongoing infection in postoperative CNS infections (22). In this case, the patient had a significantly elevated CSF leukocyte 11 days after ventricular meningioma surgery. We took CSF for culture and mNGS testing at the same time. The mNGS results reported MDR *A. baumannii* 2 days later, while the CSF culture results returned MDR *A. baumannii* 5 days later. Interestingly, the drug resistance genes by mNGS and the phenotypes of the drug susceptibility test were essentially identical. This demonstrates the ability of mNGS to detect pathogens and predict drug resistance factors rapidly and accurately. The progressive decrease of specific reads of the pathogen detected by mNGS during the course also indirectly indicated the effectiveness of adjusted anti-infective therapy. It is worth noting that the third CSF cultures were negative compared to mNGS, possibly since cultures are more susceptible to antibiotic use.

Interestingly, the third blood mNGS on the twenty-seventh postoperative day was positive for *A. baumannii*, which was negative in previous blood mNGS detections. One possible reason is that the blood-brain barrier was damaged due to the patient's multiple open cranial surgeries or other factors, such as inflammation and tumors (18). Pathogens enter the blood through the damaged blood-brain barrier. However, the patient had no obvious signs of systemic infection by *A. baumannii*. It may be because the antibiotics given to the patient also inhibited the growth of *A. baumannii* in the blood. Another possible reason is that the nucleic acids of the pathogen entered the blood *via* the damaged blood-brain barrier. Nevertheless, this case reminds us real-time monitoring of blood pathogens in patients after craniotomy is necessary.

Intravenous and intrathecal polymyxin and tigecycline are often the choices of MDR/XDR *A. baumannii* CNS infection (23). Maintaining patency of CSF drainage and ventricular lavage is also crucial in the treatment of *A. baumannii* intracranial infections. Intrathecal injection of drugs combined with continuous drainage of the lumbar pool is more clinically effective than treatment with intravenous antibiotics only (24). In this case, the patient was treated with both intrathecal polymyxin and tigecycline injections immediately after the pathogenetic evidence was clarified.

In conclusion, infection remains one of the most important complications after ventricular meningioma surgery, seriously affecting the patient's prognosis. mNGS allows early and accurate identification of the responsible causative agent and prediction of drug resistance or virulence factors. In addition, the application of multiple mNGS tests during the course can monitor the state of illness and assess the treatment efficacy.

## Data availability statement

The datasets for this article are not publicly available due to concerns regarding participant/patient anonymity. Requests to access the datasets should be directed to the corresponding author.

## Ethics statement

The studies involving human participants were reviewed and approved by IRB of Beijing Tiantan Hospital, Capital Medical University. Written informed consent to participate in this study was provided by the participants' legal guardian/next of kin. Written informed consent was obtained from the minor(s)' legal guardian/next of kin for the publication of any potentially identifiable images or data included in this article.

## Author contributions

YT was involved in the collection of data. YT, LZ, and J-XZ were involved in drafting and editing the manuscript. HX performed the mNGS. All authors have read and approved the manuscript.

## Funding

This work was supported by the Beijing Municipal Commission of Science and Technology (Grant No. Z201100005520050). Funders had no role in this case, data collection and analysis, publication decisions, or manuscript preparation.

## Conflict of interest

Author HX was employed by Hugobiotech Co., Ltd. The remaining authors declare that the research was conducted in the absence of any commercial or financial relationships that could be construed as a potential conflict of interest.

## Publisher's note

All claims expressed in this article are solely those of the authors and do not necessarily represent those of their affiliated organizations, or those of the publisher, the editors and the reviewers. Any product that may be evaluated in this article, or claim that may be made by its manufacturer, is not guaranteed or endorsed by the publisher.

## Abbreviations

CNS: Central nervous system
mNGS: Metagenomic next-generation sequencing
CSF: Cerebrospinal fluid
*A. baumannii*: *Acinetobacter baumannii*
MDR: Multidrug-resistant
XDR: Extensive drug-resistant
MRI: Magnetic resonance imaging
CPA: Cerebellopontine angle
CT: Computed Tomography
WBC: White blood cell
GR: Granulocyte
ESI-MS: Electro Spray Ionization-Mass Spectroscopy
PCR: Polymerase chain reaction.
