# Supplementary figures and images for: Detection of multidrug-resistant Acinetobacter baumannii by metagenomic next-generation sequencing in central nervous system infection after neurosurgery: A case report
Source: Front Public Health. 2022 Oct 21;10:1028920. doi: 10.3389/fpubh.2022.1028920 (PMC9634161; doi:10.3389/fpubh.2022.1028920)

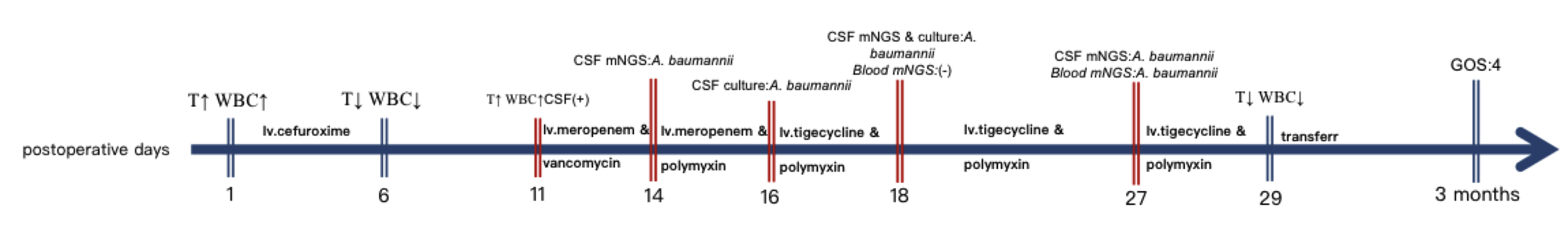

Supplement: Supplementary file 1 [file Image_1.TIFF]
